# Threatened Neotropical Primates Harbor Genetically Diverse Hemoplasmas, Including Putative Novel Genotypes and Previously Unrecognized Host Associations

**DOI:** 10.3390/microorganisms14102292

**Published:** 2026-10-09

**Authors:** Rodrigo Camêlo, Tamires Ataides Silva, Alcides Pissinatti, Silvia Moreira, Daniel Ubiali, Andresa Guimarães, Cristiane Divan Baldani, Ana Carolina Borsanelli, Filipe Dantas-Torres, Felipe Krawczak

**Affiliations:** 1 Setor de Medicina Veterinária Preventiva, Escola de Veterinária e Zootecnia, Universidade Federal de Goiás—UFG, Goiânia 74690-900, GO, Brazil; rodrigo.camelo@discente.ufg.br (R.C.); tamires_ataides@discente.ufg.br (T.A.S.); anaborsanelli@ufg.br (A.C.B.); 2 Centro de Primatologia do Rio de Janeiro, Instituto Estadual do Ambiente, Guapimirim 25940-000, RJ, Brazil; alcidespissinatti@gmail.com (A.P.); silviabm.inea@gmail.com (S.M.); 3 Instituto de Veterinária, Universidade Federal Rural do Rio de Janeiro, Seropédica 23890-000, RJ, Brazil; danielubiali@hotmail.com (D.U.); andresaguimaraes02@yahoo.com.br (A.G.); crisbaldani@gmail.com (C.D.B.); 4 Departamento de Imunologia, Centro de Pesquisa Aggeu Magalhães, Fundação Oswaldo Cruz, Recife 50670-420, PE, Brazil; fdtvet@gmail.com

**Keywords:** hemotropic mycoplasmas, hemoparasites, one health, conservation

## Abstract

Hemoplasmas are hemotropic mycoplasmas that infect erythrocytes and may cause hemolytic anemia in mammalian hosts. However, information on their occurrence and genetic diversity in threatened Neotropical primates remains scarce. This study investigated the molecular occurrence of hemoplasmas in captive-born and free-ranging born Neotropical primates at a conservation center in southeastern Brazil. Blood samples from 70 non-human primates (NHPs) representing six species were screened by qPCR and cPCR targeting the 16S rRNA gene. Hemoplasma DNA was detected in 17.1% (12/70) of individuals, with positivity in *Alouatta guariba*, *Leontopithecus chrysomelas*, *Leontopithecus rosalia*, and *Sapajus robustus*; no positive cases were detected in *Sapajus xanthosternos* or hybrid *Sapajus* spp. No statistically significant associations were observed between hemoplasma positivity and sex, origin, or hematological abnormalities, except for thrombocytosis, which was statistically associated with hemoplasma-negative animals. Phylogenetic analyses revealed the circulation of at least four distinct hemoplasma lineages, including two putative novel genotypes closely related to *Candidatus* Mycoplasma haematomacacae. These findings expand the known host range of hemoplasmas in Brazil and provide the first molecular report of hemoplasma DNA in *A. guariba*, *L. rosalia*, and *S. robustus*. The results highlight the importance of molecular surveillance to support health management and conservation strategies for threatened captive NHPs.

## 1. Introduction

Neotropical primates are Platyrrhine non-human primates (NHPs) native to the New World, with a geographic range extending from southern Mexico to northern Argentina [1]. Brazil harbors the greatest diversity of Neotropical primates worldwide, with approximately 151 species, nearly 40% of which are currently threatened with extinction, including the yellow-breasted capuchin (*Sapajus xanthosternos*), the golden lion tamarin (*Leontopithecus rosalia*), the brown howler monkey (*Alouatta guariba*), the crested capuchin (*Sapajus robustus*), and the golden-headed lion tamarin (*Leontopithecus chrysomelas*) [2,3]. In this context, ex situ conservation programs are essential for maintaining threatened NHP populations; however, captive environments may facilitate pathogen transmission due to increased host density and prolonged animal maintenance [4,5]. Recent studies of captive mammals in Brazil have documented the circulation of multiple vector-borne pathogens under ex situ conditions [5], underscoring the importance of pathogen surveillance in wildlife conservation centers.

From a One Health perspective, the drivers of NHP population decline are closely linked to habitat fragmentation, hunting, and infectious diseases [6,7]. These factors not only threaten wildlife conservation but may also facilitate the transmission of zoonotic agents [8,9]. A wide variety of hemoparasites have been reported in Neotropical primates, including *Leishmania* sp., *Trypanosoma* sp., *Plasmodium* sp., and *Mycoplasma* spp. [10,11,12,13,14]. Hemotropic mycoplasmas, commonly known as hemoplasmas, are Gram-negative, wall-less, pericellular bacteria that infect the surface of erythrocytes [15,16]. In mammalian hosts, hemoplasma infections are often subclinical but may cause hemolytic anemia, especially in immunocompromised animals [16,17]. Although some hemoplasma species have been reported as potentially zoonotic, such as *Candidatus* Mycoplasma haemohominis and hemoplasmas closely related to *Mycoplasma ovis* and *Mycoplasma suis*, there are currently no reports of hemoplasma genotypes found in NHPs that have infected humans [14,18,19].

Among NHPs, provisional hemoplasma species reported to date include *Candidatus* Mycoplasma aoti, *Candidatus* Mycoplasma kahanei, and *Candidatus* Mycoplasma haematomacacae, along with putative novel hemoplasmas (*Mycoplasma* spp.) phylogenetically related to *Ca.* M. kahanei and *Ca.* M. haematomacacae [17,20]. Despite growing recognition of hemoparasites in Neotropical primates, information on the epidemiology, host range, and health implications of hemoplasmas in threatened Neotropical primates under captive management remains scarce.

Therefore, investigating hemoplasma occurrence in Neotropical primates is essential to advance understanding of host–pathogen interactions and to support wildlife health surveillance and conservation strategies for threatened captive species. This study aimed to investigate the occurrence and molecular diversity of hemoplasmas in captive-born and free-ranging born Neotropical primates at a conservation center in Brazil. To the best of the author’s knowledge, this is the first molecular report of hemoplasmas in *L. rosalia*, *A. guariba*, and *S. robustus*.

## 2. Materials and Methods

### 2.1. Ethical Approval

This study was approved by the Institutional Animal Care and Use Committee (CEUA) of the Federal University of Goiás (protocol 103/24). All animal procedures were conducted in accordance with ethical standards for animal experimentation.

### 2.2. Study Area

This study was conducted in the state of Rio de Janeiro, southeastern Brazil, within the Atlantic Forest biome, a globally recognized biodiversity hotspot known for its high species richness and endemism. The region has a tropical climate, with high annual rainfall and temperature variability shaped by coastal and mountainous landscapes.

Sampling was conducted at the Rio de Janeiro Primatology Center (CPRJ) in the municipality of Guapimirim (22°29′ S, 42°59′ W). This conservation facility is dedicated to the rescue, rehabilitation, and management of Neotropical primates, including both captive and free-ranging individuals.

**Figure 1:**
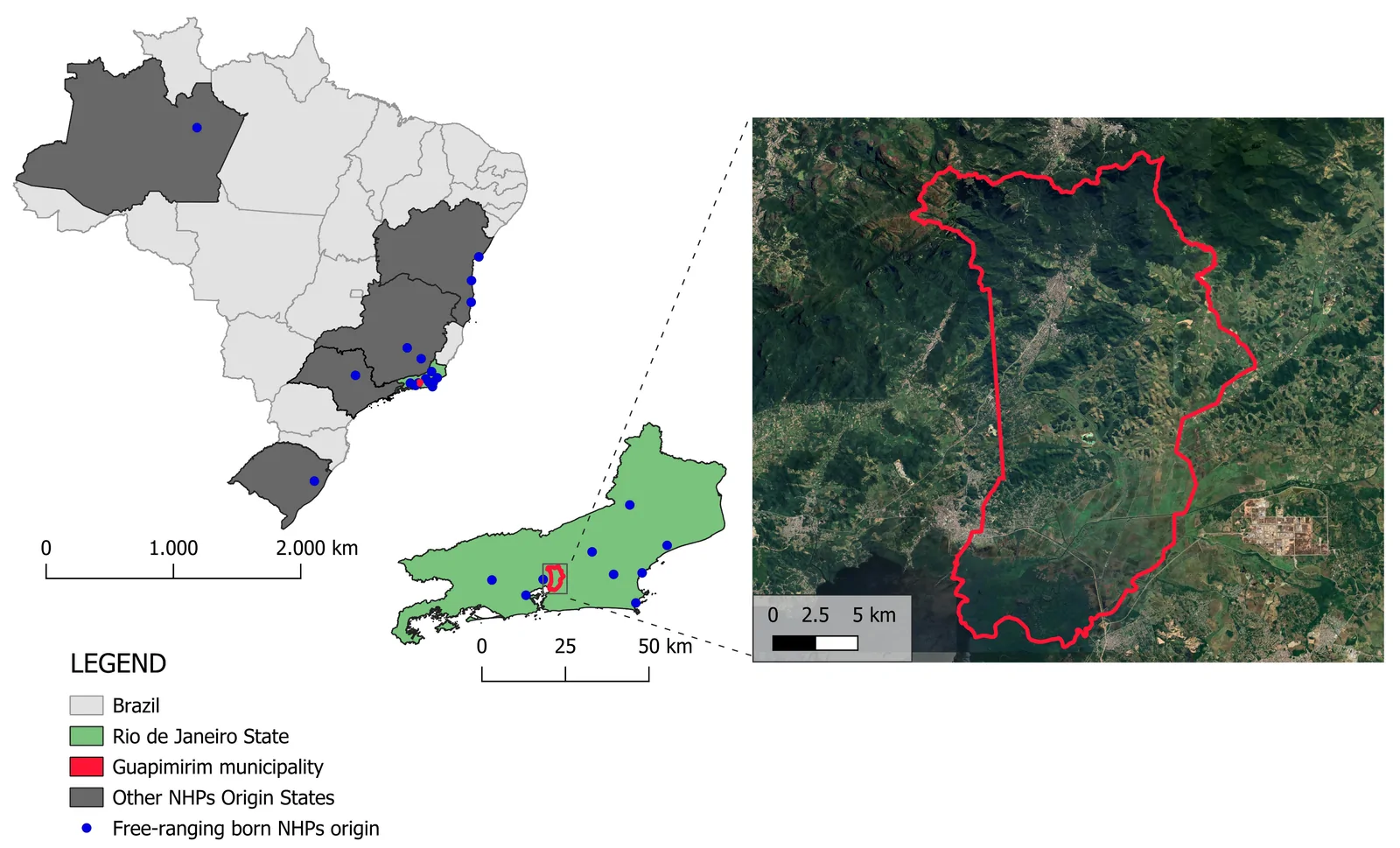
Geographic origins of NHPs included in this study and the Guapimirim municipality, where CPRJ is located.

NHPs originated from multiple municipalities in Rio de Janeiro State, with additional individuals from other Brazilian states (Figure 1). Within Rio de Janeiro, municipalities included Paracambi (n = 1), Carapebus (n = 1), Cabo Frio (n = 1), Itaocara (n = 1), Nova Friburgo (n = 1), Magé (n = 1), Duque de Caxias (n = 1), Rio das Ostras (n = 1), Silva Jardim (n = 3), and the municipality of Rio de Janeiro (n = 1). Additionally, animals were received from other states, including Minas Gerais [Belo Horizonte (n = 2) and Viçosa (n = 1)], São Paulo [São Carlos (n = 1)], Bahia [Salvador (n = 1), Porto Seguro (n = 1), and Ilhéus (n = 1)], Rio Grande do Sul [Viamão (n = 1)], and Amazonas [Manaus (n = 1)]. A total of 48 captive-born individuals originated from CPRJ, while one individual had an unidentified geographical origin.

### 2.3. Sample Collection

In May 2025, blood samples (n = 70) were collected by convenience from 70 captive NHPs representing six species at CPRJ in Guapimirim, Rio de Janeiro, southeastern Brazil. The sampled species included 24 yellow-breasted capuchins (*S. xanthosternos*), 10 brown howler monkeys (*A. guariba*), 10 golden-headed lion tamarins (*L. chrysomelas*), 10 golden lion tamarins (*L. rosalia*), 13 crested capuchins (*S. robustus*), and three hybrid *Sapajus* spp.

Animals were captured from their enclosures using a handheld capture net. Sampling was conducted under sedation induced by intramuscular administration of ketamine hydrochloride (10 mg/kg) and midazolam (0.5–1 mg/kg). Skin asepsis was performed on the medial aspect of the thigh with 70% ethyl alcohol. Blood samples were obtained by femoral vein or arterial puncture. Samples were collected into sterile EDTA-containing vacuum tubes and stored at −20 °C until DNA extraction.

Among the 24 yellow *S. xanthosternos*, 22 were captive-born, one was free-ranging born, and one had an unidentified origin. Of the 10 brown howler monkeys, eight were free-ranging born and two were captive-born. The golden-headed lion tamarins (*L. chrysomelas*) comprised eight captive-born individuals, one free-ranging born, and one of unidentified origin. Ten *L. rosalia* were captive-born. Among the *S. robustus*, six individuals were captive-born, five were free-ranging born, and two had an unidentified origin. Individuals from different species were housed in separate enclosures within the facility, preventing direct contact between populations.

For each sampled individual, institutional records were used to obtain species, sex, origin, capture location, and birth and entry dates into CPRJ. Entry and birth dates were used to estimate the duration of captivity prior to sample collection. Minimum and maximum captivity duration prior to blood collection were determined for each species.

### 2.4. Molecular Analysis

#### 2.4.1. DNA Extraction

Blood samples underwent genomic DNA extraction using the DNeasy Blood and Tissue Kit^®^ (Qiagen, Valencia, CA, USA) according to the manufacturer’s instructions. Each extraction batch included a negative extraction control consisting of microtubes containing ultrapure water to monitor for potential cross-contamination. Extracted DNA was stored at −20 °C until further real-time PCR (qPCR) and conventional PCR (cPCR) analyses.

#### 2.4.2. qPCR and cPCR Assays

DNA samples were initially individually screened for *Mycoplasma* spp. using a SYBR Green–based qPCR assay. Samples that tested positive were then subjected to cPCR targeting an approximately 610-base-pair (bp) fragment of the 16S rRNA gene for molecular confirmation [21]. Positive samples on the 16S rRNA cPCR were further analyzed by additional cPCR assays targeting an ~800 bp fragment of the 23S rRNA gene and a ~544 bp fragment of the *dnaK* gene [18,22]. Details on the target genes, primers, and expected fragments are reported in Table 1.

The qPCR assay was performed using a primer pair targeting a 134 bp fragment of the *Mycoplasma* spp. 16S rRNA gene [23]. Reactions were carried out in a final volume of 25 µL, containing 2.5 µL of DNA template, 12.5 µL of FastStart Universal SYBR Green Master^®^ (Rox) (Merck, Darmstadt, Germany), 1.5 µL of each primer (forward and reverse; 10 pmol), and 7.0 µL of nuclease-free ultrapure water.

Samples were considered positive if they exhibited a typical exponential amplification curve, a cycle threshold (Ct) < 34, and a melting temperature (Tm) within ±1 °C of that of the positive control. The Ct cutoff was established based on Willi et al. [23]. Samples showing late amplification (Ct > 34), nonspecific fluorescence signals, or failing to meet either of the predefined Ct or Tm criteria were considered inconclusive and were further analyzed by conventional PCR (cPCR) targeting the *Mycoplasma* spp. 16S rRNA gene to confirm the presence of hemoplasma DNA. To increase confidence in our molecular findings, qPCR and cPCR results were interpreted using a paired diagnostic approach. Samples meeting all predefined qPCR positivity criteria were considered positive. Samples classified as inconclusive by qPCR were considered positive only when the corresponding 16S rRNA cPCR yielded an amplification product of the expected size (~610 bp) on agarose gel electrophoresis. Samples were considered negative by qPCR when no amplification was detected and none of the predefined criteria for an inconclusive result were met. Samples classified as negative by qPCR were not subsequently analyzed by cPCR.

Each qPCR and cPCR assay included a previously confirmed hemoplasma-positive DNA sample as the positive control and PCR-grade water (Sigma-Aldrich, St. Louis, MO, USA) as the no-template control (NTC). The SYBR Green qPCR assay used for hemoplasma screening was previously developed and validated by Willi et al. [23], with a detection limit of 1–10 target copies per reaction and amplification efficiencies ≥92%. When evaluated against species-specific TaqMan assays, the method showed a diagnostic sensitivity of 98.2% and specificity of 92.1% [23].

cPCR reactions were performed in a final volume of 20 µL, containing 2.5 µL of DNA template, 4.0 µL of FIREPol^®^ Master Mix Ready to Load (5×; 12.5 mM MgCl_2_; Solis BioDyne^®^, Tartu, Estonia), 1.0 µL of each primer (forward and reverse), and 11.5 µL of nuclease-free, ultrapure water. Amplified products were visualized by 1.5% agarose gel electrophoresis using a LED transilluminator (Kasvi^®^, São José dos Pinhais, Brazil).

To assess DNA integrity and rule out false-negative results, all *Mycoplasma* spp. negative samples were further screened by cPCR targeting a 359 bp of the mammalian cytochrome b (*cytB*) gene, using the same reaction conditions described above [24,25]. Samples that did not yield the expected product in this cPCR assay were excluded from analysis.

#### 2.4.3. Amplicon Purification, Sequencing, and Phylogenetic Analysis

PCR products were purified using the PureLink^TM^ PCR Micro Kit (Invitrogen, Carlsbad, CA, USA). Purified products were bidirectionally sequenced with the same primers used for PCR, using the BigDye Terminator v3.1 sequencing kit (Applied Biosystems, Foster City, CA, USA) on an ABI Prism 3500XL (Applied Biosystems, Foster City, CA, USA). Sanger sequencing was performed at the Instituto Aggeu Magalhães, Fundação Oswaldo Cruz (IAM-FIOCRUZ).

Sequences were trimmed (error probability limit = 0.05) and assembled using Clustal Omega in Geneious Prime^®^ 2026.1.2 (https://www.geneious.com). The resulting consensus sequences were compared to sequences available in GenBank using the Basic Local Alignment Search Tool (BLAST) for nucleotides (http://blast.ncbi.nlm.nih.gov/Blast.cgi, accessed on 19 July 2026). The consensus 16S rRNA sequences generated in this study were aligned with other sequences using Clustal Omega in Geneious Prime^®^ 2026.1.2. For this purpose, we used a dataset of 16S rRNA sequences from a previous study [26].

Phylogenetic analysis was performed in IQ-TREE 3 [27], and the best-fit evolutionary model (GTR+F+R3) was selected using ModelFinder (https://iqtree.github.io/, accessed on 19 July 2026) [28]. The evolutionary history of *Mycoplasma* spp. was inferred using maximum likelihood with 1000 ultrafast bootstrap replicates [29,30]. The consensus tree was rooted with a *Mycoplasma pneumoniae* sequence (GenBank accession number: AB680604.1). The final phylogenetic tree was edited in iTOL v7 (https://itol.embl.de, accessed on 19 July 2026). Clades A-E are defined as Narapakdeesakul et al. [26]. Clades F and G were newly designated in this study.

The nucleotide sequence type networks for the 16S rRNA sequences in the present study and reference sequences retrieved from GenBank were inferred using PopART v1.7 [31] with the TCS model [32].

### 2.5. Hematological Analysis

The CBC analysis included red blood cells (RBC) (×10^6^/µL), hemoglobin (g/dL), mean corpuscular volume (MCV) (fl), mean corpuscular hemoglobin concentration (MCHC) (%), platelets (×10^3^/µL), and white blood cells (µL), all measured using an automated cell counter (Mindray BC-30 Vet, Shenzhen Mindray Animal Medical Technology Co., LTD., Shenzhen, China).

When automated analysis was not feasible, hematological parameters were manually assessed using established protocols for wild mammal hematology [33,34]. Animals were classified as anemic if any of the RBC, MCV, or MCHC values fell below the reference range.

Blood smear evaluation was performed to assess differential leukocyte counts (eosinophils, neutrophils, monocytes) and platelet counts. Smears were stained with Giemsa and examined under optical microscopy at 1000× [35]. These analyses were conducted at the Veterinary Clinical Pathology Laboratory (LABVET) of the Federal Rural University of Rio de Janeiro (UFRRJ). Reference hematological parameters were determined according to genus-specific standards for each species included in the study [36,37,38]. The hematological parameters considered in these analyses were: anemia, leukopenia, leukocytosis, neutropenia, neutrophilia, monocytosis, thrombocytopenia, and thrombocytosis.

### 2.6. Statistical Analysis

Hemoplasma positivity was calculated as proportions with 95% exact binomial confidence intervals (Clopper–Pearson method). Associations between *Mycoplasma* spp. positivity and other variables (sex, origin, and hematological abnormalities) were evaluated using Fisher’s exact test. Effect sizes for sex, origin and hematological abnormalities were estimated as odds ratios (ORs) with corresponding 95% confidence intervals (Baptista-Pike method) in GraphPad Prism 11 (https://www.graphpad.com, accessed on 9 September 2026). A *p*-value < 0.05 was considered statistically significant.

## 3. Results

### 3.1. Molecular Detection of Hemoplasmas in NHPs

Hemoplasma DNA was detected in 17.1% (12/70; 95% CI: 9.1–28%) of evaluated Neotropical primates, using a combined qPCR/cPCR diagnostic approach targeting the 16S rRNA gene. The Ct values from positive samples ranged from 25.1 to 37.1, whereas the melting temperatures (Tm) ranged from 70.8 to 74.7 °C. Among the positive samples, one was also positive for the 23S rRNA gene, and three were positive for the *dnaK* gene. Positivity was observed in four species (Table 2). Positivity was 40% (4/10; 95% CI: 12.1–73.7%) in *A. guariba* and *L. chrysomelas*, followed by 30% (3/10; 95% CI: 6.6–65.2%) in *L. rosalia* and 7.6% (1/13; 95% CI: 0.1–36%) in *S. robustus*. No positive individuals were detected in *S. xanthosternos* (0/24; 95% CI: 0–14.2%) or in hybrid *Sapajus* spp. (0/3; 95% CI: 0–70.7%). The study population consisted of 31 males (44.3%) and 39 females (55.7%). Positivity was higher in females (23%) than in males (9.6%), but the difference was not statistically significant (*p* = 0.2043; OR = 2.8; 95% CI: 0.7–10.2).

Positive samples included four free-ranging born *A. guariba*, one free-ranging born and three captive-born *L. chrysomelas*, three captive-born *L. rosalia*, and one crested capuchin (origin not identified). No significant statistical difference was observed between free-ranging born and captive-born Neotropical primates (*p* = 0.1339; OR = 2.99, 95% CI: 0.80–9.97). All samples negative for hemoplasma DNA tested positive by cPCR targeting the *cytB* gene.

Captivity duration varied among species, ranging from approximately 0.5 to 11.9 years in brown howler monkeys, 2.5 to 20.4 years in *L. chrysomelas*, 1.6 to 15.7 years in *L. rosalia*, 1.4 to 22.1 years in *S. robustus*, 1.9 to 24.4 years in *S. xanthosternos*, and 14.8 to 16.6 years in hybrid *Sapajus* spp. The mean captivity duration for hemoplasma-positive primates was 6.7 years (range: 0.5–20.4 years).

### 3.2. Phylogenetic and BLASTn Analyses

Of 12 positive 16S rRNA samples, seven had amplicon concentrations (≥10 ng/µL) adequate for sequencing, yielding the 16S rRNA sequences in this study (PZ554739-PZ554745), which ranged from 537 to 611 bases in length. These sequences showed high identities (>96%) with sequences from an undescribed *Mycoplasma* spp. detected in *Sapajus apella* from Brazil (Table 3). Unfortunately, both 23S rRNA- and *dnaK*-positive samples could not be sequenced due to low DNA concentration (≤10 ng/µL).

**Figure 2:**
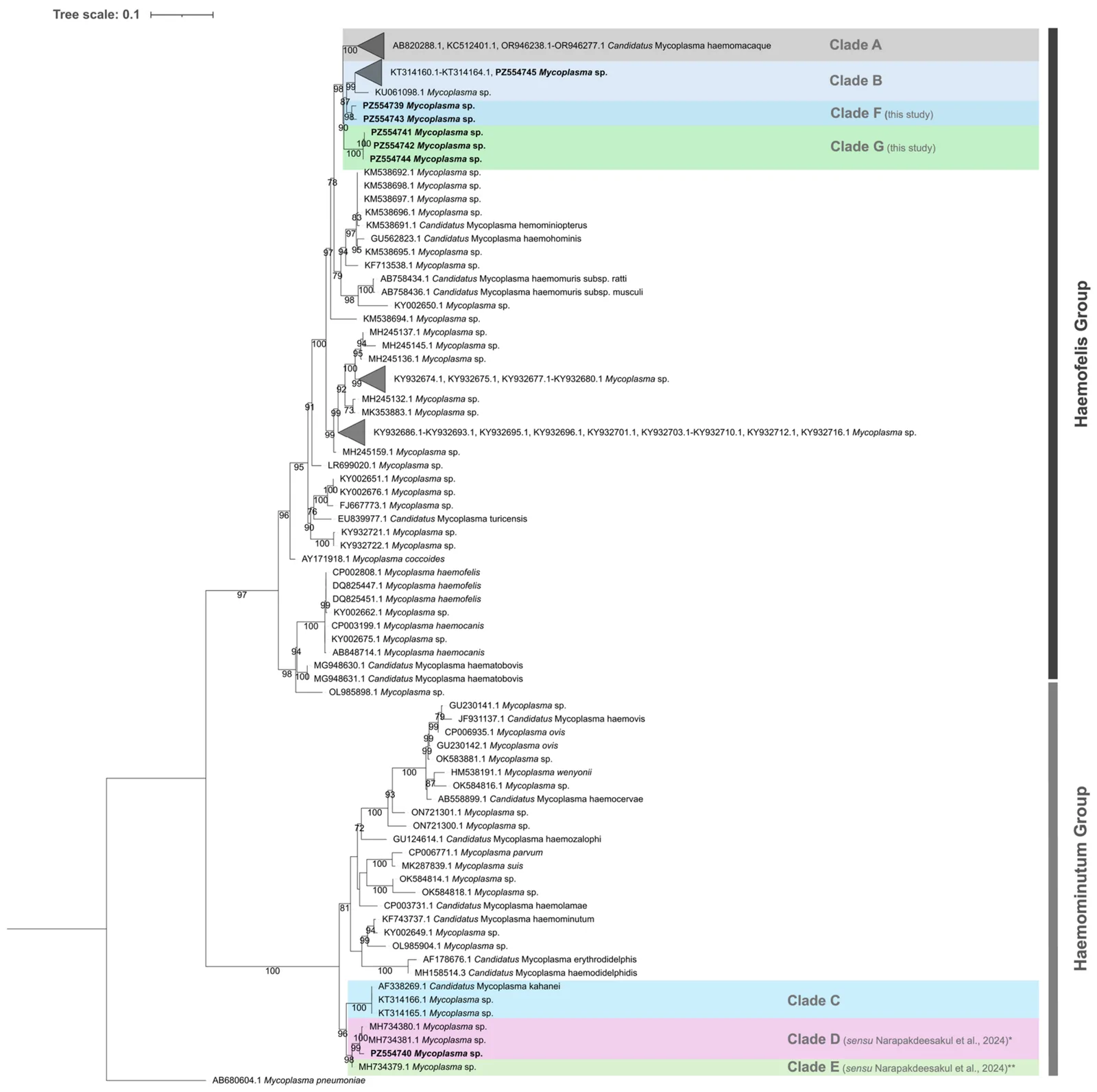
Phylogenetic analysis of 146 *Mycoplasma* spp. sequences based on an alignment of 628 nucleotide positions of the 16S rRNA gene. The tree was inferred using the maximum-likelihood method with 1000 ultrafast bootstrap replicates (bootstrap values <70 were omitted) and the GTR+F+R3 substitution model. *Mycoplasma pneumoniae* (GenBank accession number AB680604.1) served as the outgroup. Sequences generated in this study are shown in bold. * Clade D in Narapakdeesakul et al. (2024) [26] corresponds to Clade E in Mongruel et al. (2022) [20]. ** Clade E in Narapakdeesakul et al. (2024) [26] corresponds to Clade D in Mongruel et al. (2022) [20].

In the phylogenetic analysis, our sequences PZ554745 and PZ554740 clustered within Clades B and D, respectively (Figure 2). The other sequences clustered within two distinct clades in the Haemofelis group: F (PZ554739, PZ554743) and G (PZ554741, PZ554742, PZ554744).

**Figure 3:**
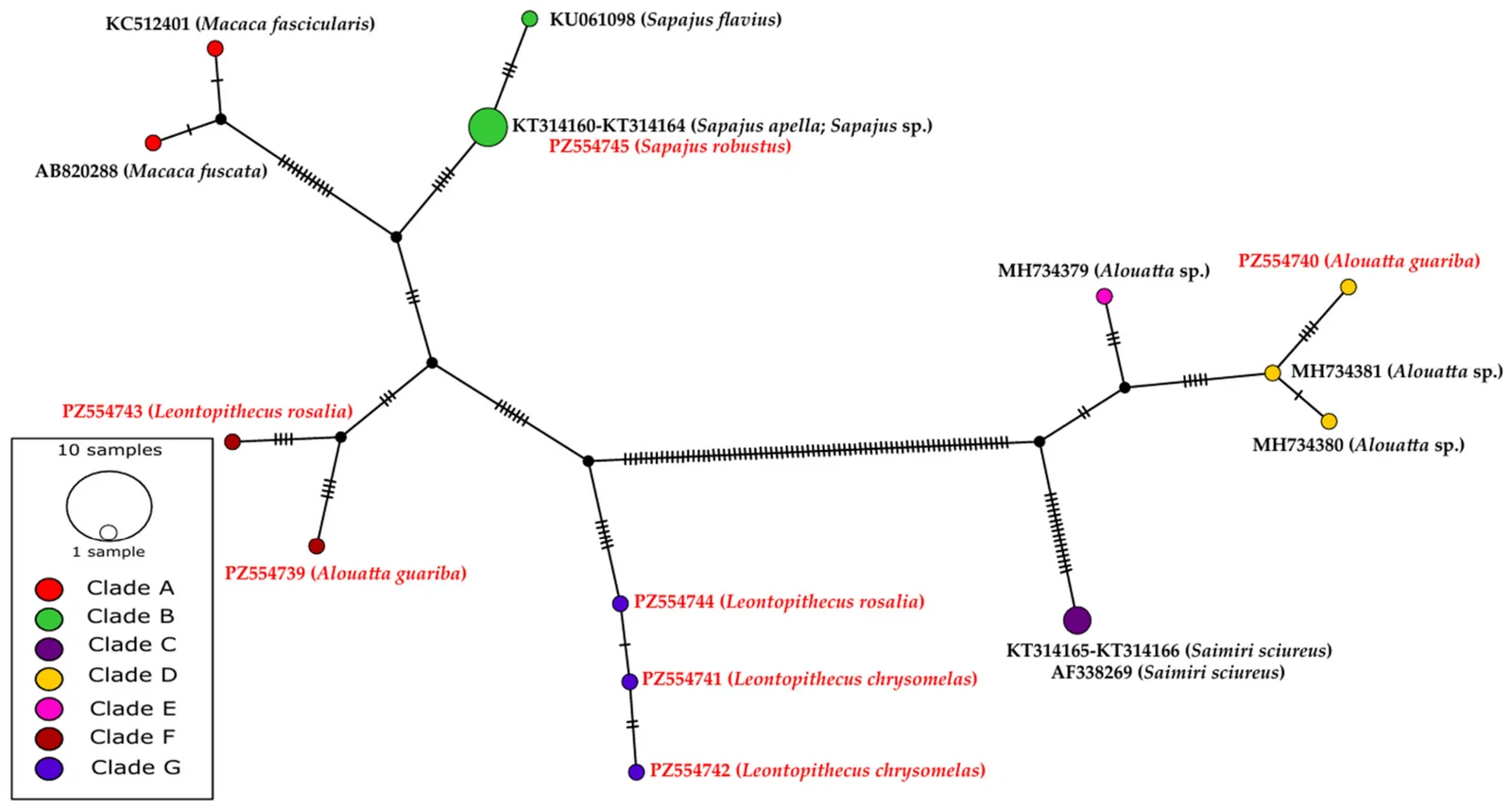
TCS haplotype network based on partial 16S rRNA sequences of hemotropic *Mycoplasma* spp. detected in Neotropical primates in this study, along with selected reference sequences from GenBank. Sequences in red are those found in this study.

Network analysis of the TCS model, based on 16S rRNA sequences from hemotropic *Mycoplasma* spp. detected in this study and reference sequences, revealed seven distinct clades (A–G), indicating genetic diversity among the analyzed sequences. The samples obtained in this study were distributed across four clades (Figure 3). Results showed a close relationship among hemoplasma lineages associated with *Ca.* M. haematomacacae (Clades A and B). Some haplotypes from this study grouped within the Haemofelis Group and formed distinct clusters (Clades F and G). In contrast, clades associated with *Ca.* M. kahanei clustered separately within the Haemominutum Group (Clade D).

### 3.3. Hematological Results

Hematological parameters were evaluated in all sampled NHPs. Hematological abnormalities by hemoplasma status are summarized in Table 4. Among hemoplasma-positive NHPs, anemia was observed in only 3/12 (25%) individuals, two brown howler monkeys and one crested capuchin. No statistically significant associations were found between hemoplasma positivity and the evaluated hematological abnormalities, except for thrombocytosis, which was statistically associated with hemoplasma-negative NHPs (*p* > 0.05).

## 4. Discussion

This study provides molecular evidence of hemoplasma infection in captive and free-ranging born Neotropical primates at a conservation center in southeastern Brazil. In Brazil, Hemoplasma DNA has previously been reported in several Neotropical primate species, including *Lagothrix lagotricha*, *A. caraya*, *S. apella*, *Saimiri sciureus*, *Saguinus midas niger*, *Alouatta* spp., *Sapajus nigritus*, *Sapajus flavius*, and *L. chrysomelas* [5,39,40,41,42,43,44]. The present findings expand the known host range of hemoplasmas and, to the authors’ knowledge, represent the first molecular detection of hemoplasmas in brown howler monkeys (*A. guariba*), golden-lion tamarins (*L. rosalia*), and crested capuchins (*S. robustus*). The detection of hemoplasmas in captive and free-ranging born individuals suggests that these organisms circulate within conservation settings.

In this study, 17.1% (12/70) of NHPs tested positive for hemoplasma DNA. To date, only four studies have investigated hemoplasmas in captive Neotropical primates in Brazil [5,41,42,43], and only two has specifically compared positivity rates across captive species [5,41]. These studies reported hemoplasma detection rates of 20.0% to 66.6% in captive NHPs, indicating that the detection rate observed here falls below the range reported for captive Neotropical primates in Brazil. However, molecular positivity comparisons should be interpreted cautiously, as hemoplasma occurrence may vary by study population, ecological factors, host density, behavioral interactions, and vector distribution [45,46]. In addition, differences in the molecular methods employed across these studies may have influenced the reported results. When comparing studies with contrasting findings, potential false-positive and false-negative results arising from differences in assay specificity and sensitivity should be considered. qPCR generally provides greater analytical sensitivity and specificity than cPCR. In the present study, we used a paired diagnostic approach (qPCR and cPCR), whereas previous studies employed only cPCR. Therefore, the lower positivity observed here may reflect a combination of these factors rather than a single determinant.

To date, reports of hemoplasmas in Brazilian Neotropical primates have been concentrated mainly in three genera, *Sapajus*, *Alouatta*, and *Saimiri* [14]. In the present study, *Mycoplasma* sp., closely related to *Ca.* M. haematomacacae, was detected in distinct host species, including *Alouatta* and *Leontopithecus*, forming newly identified clades with marked genetic separation (95.99–97.44% identity) from hemoplasmas in clades A and B within the Haemofelis group. The TCS haplotype network and phylogenetic tree analysis identified Clade F, which includes *Mycoplasma* sequences from two primate species: *A. guariba* (PZ554739) and *L. rosalia* (PZ554743). In turn, Clade G includes two sequences (PZ554741, PZ554742) from *L. chrysomelas* and one (PZ554744) from *L. rosalia*. Bonato et al. (2015) [40] reported genetic separation between *Ca.* M. haematomacacae detected in Old World monkeys from the USA and *Mycoplasma* sp. from *S. apella* from the Amazon biome. Similarly, Ramalho et al. (2017) [42] detected *Mycoplasma* sp. in captive *S. flavius* from northeastern Brazil, demonstrating close phylogenetic relationships with hemoplasmas from northeastern Brazil [40,41]. In this regard, our findings indicate the circulation of putative novel genotypes, genetically related to *Ca.* M. haematomacacae, among NHPs in southeastern Brazil, distinct from *Sapajus*-related hemoplasma genotypes in northeastern Brazil. These findings suggest that the host range and phylogenetic diversity of NHP hemoplasma lineages in Brazil may be broader than currently recognized, with regional variation in hemoplasmas. Consequently, the reported association between specific hemoplasma genotypes and particular host species may partially reflect the limited taxonomic diversity of Neotropical primates investigated to date. Further studies, including a wider range of primate species, are required to determine the extent of host specificity and genetic diversity of hemoplasmas circulating among Neotropical primates.

Positivity was detected at higher frequencies in *A. guariba* (40.0%) than in *S. robustus* (7.6%), whereas no positive individuals were identified in *S. xanthosternos* or in *Sapajus* spp. This pattern aligns with a previous study reporting higher hemoplasma positivity in captive *Alouatta* than in *Sapajus* [41]. Previous molecular studies have suggested some degree of host specificity among hemoplasma lineages [47]. This is also supported by reports on Neotropical primates, which link *Sapajus* spp. to *Ca.* M. haematomacacae and *Alouatta* spp. to *Ca.* M. kahanei lineages [43]. However, phylogenetic analysis in the present study revealed probable circulation of two distinct hemoplasma lineages in brown howler monkeys, with genotypes closely related to both *Ca.* M. haematomacacae and *Ca.* M. kahanei. Notably, our sequence PZ554740, obtained from a brown howler in southeastern Brazil was 98.20% similar to a sequence (KT824793.1) from a black howler monkey (*A. caraya*) in southern Brazil and clustered within Clade D of the Haemominutum group. It grouped with two other sequences (MH734380.1 and MH734381.1) from *Alouatta caraya* in southern Brazil. Nevertheless, the genotype detected herein also differed genetically from *Ca.* M. kahanei sequences from *S. sciureus* (AF338269) in the Amazon biome, further supporting the presence of distinct hemoplasma lineages across NHP species and biomes. Surprisingly, the other *A. guariba* hemoplasma sequence (PZ554739) showed 97.38% similarity to *Mycoplasma* sp. related to *Ca.* M. haematomacacae (Clade F), suggesting that associations between hemoplasma genotypes and NHP species may not be strict and raising the possibility of occasional interspecific transmission in captive settings. Together, these findings suggest that hemoplasmas with genetically distinct markers may circulate among *Alouatta* populations. Therefore, differences in hemoplasma positivity among NHP species are likely influenced not only by host-related factors but also by variation in exposure and circulation of distinct hemoplasma lineages across biomes and NHP populations.

The genus *Sapajus* had the largest number of tested individuals in this study. However, only one individual (*S. robustus*) tested positive, showing 100% similarity to a sequence (KT314164.1) from a tufted capuchin (*S. apella*) in northeastern Brazil [39] and clustering within Clade B of the Haemofelis group. This *Mycoplasma* sp. was also detected in a captive Marcgrave’s capuchin (*S. flavius*) from northeastern Brazil [40]. These findings suggest the presence of a genetically divergent hemoplasma lineage that infects multiple primate species across distinct regions of Brazil. Although phylogenetic analysis suggested the circulation of a hemoplasma genotype previously reported in *Sapajus* spp., the low positivity observed among *Sapajus* indicates that host-associated hemoplasma lineages do not necessarily correspond to higher detection rates.

Among *Leontopithecus* species, *L. chrysomelas* had the highest hemoplasma detection rate (40%), followed by *L. rosalia* (30%). Additional phylogenetic analysis showed that hemoplasma sequences from both *L. rosalia* and *L. chrysomelas* clustered within distinct clades identified in this study, closely related to *Mycoplasma* sp. (Clades F and G). These findings confirm the molecular occurrence of potentially distinct hemoplasma genotypes in tamarins, extend detection to captive populations of *L. chrysomelas*, and identify *L. rosalia* as an additional potential host. Previously, no studies had evaluated hemoplasma detection in captive-maintained *Leontopithecus* spp. However, Costa et al. [44] reported the molecular detection of hemoplasmas in free-ranging *L. chrysomelas*, with a positivity of 38%, slightly lower than in the present study. This finding reinforces that hemoplasma circulation can still occur in captivity, with detection rates like those in free-ranging NHPs. Several factors may contribute to hemoplasma circulation in captive settings, including host density and behavioral factors such as social structure and the frequency of physical contact among individuals of the same species [4]. Unfortunately, these variables could not be assessed in this study, underscoring the need for further research to better understand the causes of hemoplasma occurrence in ex situ programs.

Nevertheless, the present study and previous investigations on hemoplasmas in NHPs have relied primarily on 16S rRNA gene phylogenetic analyses, which limit inferences about the occurrence of novel hemoplasma species. Additional molecular characterization using more variable genetic markers, such as the 23S rRNA and RNase P RNA (*rnpB*) genes, should be incorporated into future studies to improve taxonomic resolution [26]. In the present study, amplification of the 23S rRNA and *dnaK* genes was successful in only a small number of hemoplasma-positive samples, and the resulting sequences were of insufficient quality for phylogenetic analysis. This limitation may reflect the greater genetic variability of these genes compared with the highly conserved 16S rRNA gene [26]. In addition, the limited availability of reference sequences from Neotropical primate hemoplasmas for genes other than 16S rRNA may affect primer design for genetically divergent hemoplasmas, reducing amplification efficiency and sequencing success for genetically divergent lineages.

The overall frequency of hemoplasma-positive females (23%) was higher (OR = 2.8; 95% CI: 0.7–10.2) than that of males (9.6%). Similarly, two other studies in NHPs from Thailand [17,48] reported a higher frequency of hemoplasma detection among female NHPs, although no significant statistical association between sex and positivity status was observed. Hemoplasma detection is commonly associated with male hosts, likely due to increased opportunities for direct transmission through aggressive interactions such as fighting and biting [19,41]. These contrasting findings suggest that the influence of sex on hemoplasma occurrence may vary across animal species and populations.

Positive animals were identified among both recently admitted and long-term captive individuals, with captivity periods ranging from 0.5 to 20.4 years, suggesting that prolonged captivity may not be strictly necessary for hemoplasma detection. Accurate age information was unavailable for a proportion of the evaluated NHPs, precluding a comprehensive assessment of the potential influence of age on hemoplasma infection status. Nevertheless, hemoplasma DNA was detected in an *A. guariba* six months after admission to captivity. Previous studies in laboratory-kept rhesus macaques (*Macaca mulatta*) demonstrated that hemoplasma DNA may persist for at least 10 months after initial detection [20]. Furthermore, the cross-sectional sampling design did not allow determination whether infection occurred prior to admission or was acquired after entry into the conservation facility, particularly in free-ranging born NHPs. This highlights the importance of implementing molecular screening protocols during animal intake and quarantine to reduce the risk of introducing hemoparasites into captive populations of conservation concern.

No significant hematological alterations were statistically associated with hemoplasma positivity in the present study, except thrombocytosis, which was statistically associated with hemoplasma-negative NHPs Hemoplasma-positive NHPs showed higher odds of neutrophilia, neutropenia, monocytosis, eosinophilia, and thrombocytopenia, whereas anemia, leukocytosis and thrombocytosis showed lower odds. However, since most of these associations had no statistical significance, our conclusions are limited. Anemia was observed in only three hemoplasma-positive NHPs, two *A. guariba* and one *S. robustus*. However, it remains unclear whether this finding was directly linked to hemoplasma infection. Previous studies evaluated hematological parameters in hemoplasma-positive *Alouatta*, *L. chrysomelas*, and *Sapajus* and similarly reported hemolytic anemia without statistically significant differences between positive and negative individuals [40,41,42,43,44], reinforcing the findings observed here. These findings support the conclusion that hemoplasma infection in Neotropical primates may be subclinical and that hemolytic anemia is an uncommon outcome in hemoplasma-positive NHPs [42,49]. The two anemic hemoplasma-positive *A. guariba* harbored phylogenetically distinct hemoplasma genotypes (PZ554740; PZ554739), while the anemic *S. robustus* harbored a hemoplasma genotype related to *Ca.* Mycoplasma haematomacacae (PZ554745). Although no significant hematological abnormalities have been statistically associated with *Ca.* M. haematomacacae or closely related genotypes in *Macaca* spp. and *Sapajus* spp., infections with closely related hemoplasma genotypes of *Ca.* M. kahanei in howler monkeys have been reported to cause alterations such as monocytosis, lymphocytosis, and thrombocytopenia [14,43]. Future research is needed to assess hemoplasma’s role in the development of hemolytic anemia among NHPs. Additionally, further research should investigate whether differences in hematological manifestations among hemoplasma-positive NHPs with distinct genotypes are primarily driven by putative variation in the pathogenicity of different hemoplasma lineages or by interspecies variation in host susceptibility.

Among hemoplasma-positive individuals, 41.7% (5/12) were free-ranging born, whereas 50% (6/12) were captive-born (OR = 2.99, 95% CI: 0.80–9.97), with no statistically significant association between origin and positivity status. Although captive NHPs may typically be less exposed to ectoparasites than wild NHPs and benefit from grooming, which aids ectoparasite removal [46,50], these factors appear insufficient to prevent hemoplasma circulation in captive settings. Individuals from different species in this study were housed in separate enclosures, suggesting that direct interspecific transmission was unlikely. This observation suggests that other transmission routes may contribute to the maintenance and spread of hemoplasma within captive populations. Hemoplasma infections are frequently associated with ectoparasite-mediated transmission [51,52], but accumulating evidence suggests that hemoplasma transmission is likely multimodal [19,48]. However, the phylogenetic similarity observed among hemoplasma sequences from different host species could not distinguish between interspecific transmission, circulation of shared ancestral lineages, exposure to common vectors, or independent acquisition of closely related hemoplasmas. Since the present study did not assess the presence of hemoplasma DNA in potential arthropod vectors our conclusions remain only hypothetical. Therefore, interspecific transmission should be considered only as a possibility for hemoplasma occurrence in this study. Together, these findings underscore the need for broader epidemiological studies to clarify the mechanisms underlying hemoplasma transmission in Neotropical primates.

All animals were sampled by convenience at a single conservation center, with a relatively small number of individuals within some NHP species, which may limit the extrapolation of our findings to broader NHP populations. Additionally, the small number of positive animals may have limited the statistical power to detect associations between hemoplasma positivity, host characteristics, and hematological abnormalities. Differences in the distribution of animal origin among host species may also have limited direct comparisons between captive-born and free-ranging-born individuals. For instance, all *L. rosalia* individuals were captive-born, whereas eight of ten *A. guariba* individuals were free-ranging-born. Therefore, the observed patterns associated with animal origin may have been partially influenced by species-specific differences rather than by origin itself. Consequently, the absence of significant statistical associations between NHP characteristics and hemoplasma positivity should be interpreted cautiously. Nevertheless, our study evaluated a relatively large number of threatened NHPs from Brazil’s largest primatology conservation center, and future studies with more balanced sampling across host species and origin categories are needed to better assess the potential influence of animal origin on hemoplasma occurrence in captive NHPs.

## 5. Conclusions

This study provides the first molecular evidence of hemoplasma infection in *A. guariba*, *L. rosalia*, and *S. robustus*, with additional detection in *L. chrysomelas* held at a conservation center. Phylogenetic analysis indicated the circulation of at least four distinct hemoplasma lineages across multiple NHP species, including *Mycoplasma* sp. closely related to *Ca.* M. haematomacacae and another *Mycoplasma* sp. closely related to *Ca.* M. kahanei, thereby expanding current knowledge of hemoplasma diversity and host range in Neotropical primates. This underscores the importance of broader hemoparasite screening programs, including agents frequently associated with subclinical infections, such as hemoplasmas. The identification of multiple potentially susceptible NHP host species underscores the need for routine wildlife health surveillance to prevent the introduction and spread of potentially pathogenic agents among endangered populations. Overall, these findings highlight the value of integrating molecular pathogen monitoring into conservation and wildlife management programs to support species preservation objectives. Future studies using additional molecular markers are also needed to better resolve the taxonomic diversity and evolutionary relationships of hemoplasmas circulating among Neotropical primates.

## Figures and Tables

**Table 1:** Primers used for *Mycoplasma* spp. cPCR and qPCR assays.

Target Genes	Primer Sequences	Fragment Size	References
cPCR Assays			
16S rRNA	HBT-F (F) 5′-ATACGGCCCATATTCCTACG-3′ HBT-R (R) 5′-TGCTCCACCACTTGTTCA-3′	~610 bp	Criado-Fornelio et al., 2003 [21]
23S rRNA	5′-TGAGGGAAAGAGCCCAGAC-3′ 5′-GGACAGAATTTACCTGACAAGG-3′	~800 bp	Mongruel et al., 2020 [22]
*dnaK*	5′-GGGTGGAGATGATTGAGACCA-3′ 5′-AGCCACCCCTCCTAGAGTTT-3′	~544 bp	Descloux et al., 2021 [18]
qPCR Assays			
16S rRNA	SYBR_For (F) 5′-AGCAATRCCATGTGAACGATGAA-3′ SYBR_Rev1 (R) 5′-TGGCACATAGTTWGCTGTCACTT-3′	134 bp	Willi et al., 2009 [23]

**Table 2:** Number and species of NHPs positive for hemoplasma DNA in this study and their origins.

NHP Species	No. of NHPs (%)	No. of Free-Ranging Born (%)	No. of Captive-Born (%)	No. of Unidentified Origin (%)	No. of Positives (%)
*Sapajus xanthosternos*	24 (34.2%)	1 (4.1%)	22 (91.6%)	1 (4.1%)	0
*Alouatta guariba*	10 (14.2%)	8 (80%)	2 (20%)	0	4 (40%)
*Leontopithecus chrysomelas*	10 (14.2%)	1 (10%)	8 (80%)	1 (10%)	4 (40%)
*Leontopithecus rosalia*	10 (14.2%)	0	10 (100%)	0	3 (30%)
*Sapajus robustus*	13 (18.5%)	5 (38.4%)	6 (46.1%)	2 (15.3%)	1 (7.6%)
hybrid *Sapajus* spp.	3 (4.2%)	2 (66.6%)	1 (33.3%)	0	0
Total	70 (100%)	17 (24.2%)	49 (70%)	4 (5.7%)	12 (17.1%)

NHPs: Non-human Primates; No.: Number.

**Table 3:** BLASTn results of the sequences of the 16S rRNA gene for mycoplasmas detected in NHPs from the southeastern region of Brazil.

Sample No. (GenBank Accession) NHP Species	Size (bp)	Identity	Query Cover (%) /E-Value	Sequence Best Match Location/Host GenBank Accession
Sample 25 PZ554739 *Alouatta guariba*	573	97.38%	100%/0.0	*Mycoplasma* sp. *Sapajus* apella/Brazil KT314164.1
Sample 29 PZ554740 *A. guariba*	611	98.20%	100%/0.0	*Mycoplasma* sp. *Alouatta caraya*/Brazil KT824793.1
Sample 36 PZ554741 *Leontopithecus chrysomelas*	570	96.15%	100%/0.0	*Mycoplasma* sp. *S. apella*/Brazil KT314164.1
Sample 41 PZ554742 *L. chrysomelas*	580	95.99%	99%/0.0	*Mycoplasma* sp. *S. apella*/Brazil KT314164.1
Sample 49 PZ554743 *Leontopithecus rosalia*	585	97.44%	100%/0.0	*Mycoplasma* sp. *S. apella*/Brazil KT314160
Sample 50 PZ554744 *L. rosalia*	577	96.37%	100%/0.0	*Mycoplasma* sp. *S. apella*/Brazil KT314164.1
Sample 61 PZ554745 *Sapajus robustus*	577	100%	100%/0.0	*Mycoplasma* sp. *S. apella*/Brazil KT314164.1

**Table 4:** Hematological findings in NHPs and their association with hemoplasma-positive rates.

Hematological Alterations	Positive Animals/ Hematological Alterations (%)	Negative Animals/ Hematological Alterations (%)	*p*-Value	Odds Ratio (95% CI)
Anemia	3/12 (25%)	20/58 (34.4%)	0.7383	0.6 (0.1–2.3)
Leukopenia	0/12 (0%)	0/58 (0%)	1.000	NE
Leukocytosis	1/12 (8.3%)	5/58 (8.6%)	1.000	0.9 (0.1–6.8)
Neutrophilia	1/12 (8.3%)	3/58 (5.1%)	0.5373	1.6 (0.1–12.0)
Neutropenia	1/12 (8.3%)	1/58 (1.7%)	0.3155	5.1 (0.2–99.6)
Monocytosis	1/12 (8.3%)	3/58 (5.1%)	0.5373	1.6 (0.1–12.0)
Eosinophilia	5/12 (41.6%)	10/58 (17.2%)	0.1145	3.4 (0.9–12.6)
Thrombocytopenia	1/12 (8.3%)	4/58 (6.8%)	1.000	1.2 (0.1–8.9)
Thrombocytosis	2/12 (16.6%)	32/58 (55.1%)	0.0242	0.1 (0.1–0.7)

ORs represent the odds of each hematological alteration among hemoplasma-positive versus hemoplasma-negative NHPs. Odds ratios not estimated due to zero values are indicated as “NE”.

## Data Availability

The original contributions presented in this study are included in the article/Supplementary Materials. Further inquiries can be directed to the corresponding author.

## Supplementary Materials

The following supporting information can be downloaded at: https://www.mdpi.com/article/10.3390/microorganisms14102292/s1, File S1: Animal Characteristics, Molecular Data, and Hematological Results..

## Abbreviations

The following abbreviations are used in this manuscript:

NHP	Non-Human Primate
CEUA	Institutional Animal Care and Use Committee
CPRJ	Rio de Janeiro Primatology Center
qPCR	Real-time Polymerase Chain Reaction
cPCR	Conventional Polymerase Chain Reaction
Ct	Cycle Threshold
Tm	Melting Temperature
